# Microsatellite diversity among the primitive tribes of India

**DOI:** 10.4103/0971-6866.60187

**Published:** 2009

**Authors:** Malay B. Mukherjee, V. Tripathy, R. B. Colah, P. K. Solanki, K. Ghosh, B. M. Reddy, D. Mohanty

**Affiliations:** Department of Hemato-Genetics, National Institute of Immunohaematology, 13^th^ Floor, NMS Building, KEM Hospital Campus, Parel, Mumbai - 400 012, India; 1 Molecular Anthropology Group, Biological Anthropology Unit, Indian Statistical Institute, Street No. 8, Habsiguda, Hyderabad - 500 007, India

**Keywords:** Genetic diversity, India, microsatellite, polymorphism STRs, tribes

## Abstract

The present study was undertaken to determine the extent of diversity at 12 microsatellite short tandem repeat (STR) loci in seven primitive tribal populations of India with diverse linguistic and geographic backgrounds. DNA samples of 160 unrelated individuals were analyzed for 12 STR loci by multiplex polymerase chain reaction (PCR). Gene diversity analysis suggested that the average heterozygosity was uniformly high ( >0.7) in these groups and varied from 0.705 to 0.794. The Hardy-Weinberg equilibrium analysis revealed that these populations were in genetic equilibrium at almost all the loci. The overall G_ST_ value was high (G_ST_ = 0.051; range between 0.026 and 0.098 among the loci), reflecting the degree of differentiation/heterogeneity of seven populations studied for these loci. The cluster analysis and multidimensional scaling of genetic distances reveal two broad clusters of populations, besides Moolu Kurumba maintaining their distinct genetic identity vis-à-vis other populations. The genetic affinity for the three tribes of the Indo-European family could be explained based on geography and Language but not for the four Dravidian tribes as reflected by the NJT and MDS plots. For the overall data, the insignificant MANTEL correlations between genetic, linguistic and geographic distances suggest that the genetic variation among these tribes is not patterned along geographic and/or linguistic lines.

## Introduction

The most remarkable feature of the Indian population structure is the clear division of its population into strictly defined endogamous castes, tribes and religious groups. With the exception of Africa, India harbors more genetic diversity than other comparable global regions. It is generally believed that the tribal people, who constitute 8.2% of the total population (2001 census of India), are the original inhabitants of India. The total number of tribal groups is estimated to be 461, who speak about 750 dialects that belong to one of the four language groups, Austro-Asiatic, Indo-Europeans, Dravidian and Tibeto-Burman.[1,2] It is possible that populations living in close geographic proximity are more likely to exchange genes, thereby enhancing genetic similarity, despite the fact that these populations may not belong to the same sociocultural stratum.

Studies based on autosomal markers (STRs) have suggested that genetic distances are correlated with geographical distances among the Indian populations.[3-5] Indeed, geographical clines have been reported for traditional genetic markers like ABO allele frequencies.[6] Nevertheless, clines for other genetic markers are observed to be restricted to very small radius, not over long distances, as reflected by the autocorrelation analyses of traditional genetic markers and quantitative variables like anthropometry and dermal ridge counts.[7,8] This has been ascribed to the unique Indian population structure, characterized by strict endogamy of the castes and tribes, which fits the kind of island model rather than the isolation by distance model of population structure. It has also been argued that tribes belonging to different language families represent different genetic lineages; hence, they are genetically different.[9] Based on autosomal markers, Roychoudury *et al*. reported close genetic affinity for populations from similar linguistic backgrounds.[10] The present study was undertaken to determine the extent of genetic variation based on 12 STR loci among seven primitive tribal populations of India, belonging to the same ethnic group traditionally described as Australoid. These tribes speak languages belonging to two different linguistic families and are widely separated geographically.

## Materials and Methods

**Figure 1:**
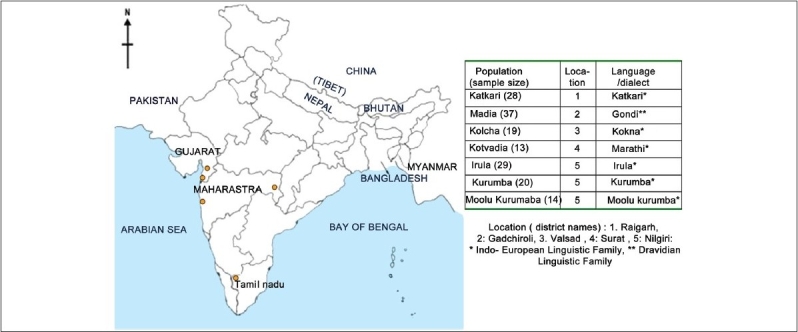
Map of India showing the locations from where samples were collected along with information on languages spoken by these tribes

The location of the study populations along with the linguistic background and sample sizes are presented in Figure 1. The Indo-European language-speaking tribes Kolcha, Kotvadia and Katkari are from the neighboring states of Gujarat and Maharashtra. The three Dravidian language-speaking tribes Irula, Kurumba and Moolu Kurumba are from the Nilgiri district of Tamil Nadu whereas Madia are a group belonging to the Dravidian language family and are from the state of Maharashtra, which is predominantly inhabited by populations speaking Indo-European languages. Interestingly, the Gondi language spoken by Madia has an Indo-European script in the north and a Dravidian script in the south.

Five to 10 ml of blood was collected in EDTA after informed consent from 160 unrelated individuals belonging to the seven tribal groups. DNA was isolated from the leucocytes by using standard protocols.[11] Twelve dinucleotide microsatellite STR loci (D12S83, D13S218, D12S78, D13S217, D12S1659, D13S285, D13S170, D12S1723, D13S175, D13S263, D12S1617 and D12S346) were analyzed by multiplex PCR using commercially available ABI Prism Linkage Mapping sets V2.5 kits (Applied Biosystems, Foster city, California, USA). The samples were run on the ABI Prism 310 Genetic analyzer (Applied Biosystems) using the Gene Scan program. The resultant data analysis was carried out using the Genotyper software. Alleles at 12 loci were designated by repeat numbers.

Allele frequencies at each locus were calculated by a simple gene counting method.[12] The Hardy-Weinberg equilibrium for each locus was tested by the Exact test, which was performed using software Arlequin version 3.0.[13] Nei's coefficient of gene differentiation (GST), which is based on mean heterozygosity within populations (H_S_) and mean heterozygosity for the total sample (H_T_) {GST = 1- (HS/HT)}, was calculated using the software Dispan.[14] Pair-wise genetic distances between populations (DA distance) following Nei *et al*.[15] and a phylogenetic tree based on the neighbor-joining (NJ) method proposed by Saitou and Nei[16] were constructed using the software Dispan. Arlequin software version 3.0 was used for the analysis of molecular variance (AMOVA). Powerstats V12 (www.promega.com/geneticidtools/powerstats/PowerStatsV12.xls) was used to calculate the power of discrimination (PD). Multidimensional scaling analysis based on DA distances was performed with SPSS version 10.0. Mantel correlations for matrix correspondence and partial correlations were obtained between the genetic, geographic and linguistic distance matrices with the software F-Stat version 2.9.3.2 available at http://www2.unil.ch/popgen/softwares/fstat.htm. The geographic distance between places was computed based on geographic coordinates of the place from where the samples were collected. Linguistic distance for the correlation analyses was based on the linguistic trees,[17] considering different languages spoken by these tribes within each of the linguistic families. The scheme used was one unit distance between populations separated at each branch nodes.

## Results

Allele frequencies at 12 STR loci among the seven tribal groups showed the presence of the same common alleles and, in a majority of the cases, the most predominant allele was the same, with a variable frequency. The observed heterozygosity at each locus and the average heterozygosity over all the loci for each of the study populations are given in Table 1. The average heterozygosity that indicates the degree of within-population variation is uniformly high (>0.7), varying from 0.705 to 0.795. Among the different loci, D13S263 showed the highest level of heterozygosity in the populations (0.643-0.923) and D12S1659 the lowest (0.300-0.678). The Hardy-Weinberg equilibrium analysis revealed that these populations are in genetic equilibrium in almost all the loci studied. Gene diversity analysis for individual loci and for all the loci taken together is presented in Table 2. The coefficient of gene differentiation among the populations is variable across the loci. The overall extent of genetic differentiation among the seven groups is high (G_ST_ = 0.051). However, there is considerable heterogeneity in the degree of differentiation at different loci, high (9.8%) in the case of D13S175 and low (2.6%) in the case of D13S170. The PD is an index of the power of a particular locus to discriminate individuals in a population and the results suggest that most of these loci show a high value of this index.

**Table 1:** Observed and average heterozygosities at different loci in seven tribal groups

Locus	Katkaris (n = 56)	Madias (n = 74)	Irulas (n = 58)	Kurumbas (n = 40)	Moolu kurumbas (n = 28)	Kolchas (n = 38)	Kotvadias (n = 26)
D12S83	0.750	0.865	0.793	0.650	0.714	0.894	0.538
D13S218	0.785	0.675	0.655	0.450	0.357	0.789	0.923
D12S78	0.928	0.838	0.793	0.900	0.929	0.894	0.846
D13S217	0.928	0.838	0.827	0.850	0.357	0.684	0.846
D12S1659	0.678	0.432	0.551	0.300	0.500	0.368	0.384
D13S285	0.857	0.757	0.862	0.800	0.990	0.789	0.846
D13S170	0.821	0.892	0.586	0.700	0.928	0.947	0.692
D12S1723	0.643	0.784	0.586	0.800	0.714	0.684	0.384
D13S175	0.607	0.649	0.689	0.500	0.571	0.684	0.538
D13S263	0.821	0.810	0.896	0.700	0.643	0.684	0.923
D12S1617	0.821	0.729	0.827	0.900	0.928	0.842	0.692
D12S346	0.893	0.838	0.862	0.950	0.857	0.789	0.846
Average	0.794	0.759	0.744	0.708	0.708	0.754	0.705

*n* = Number of chromosomes studied

**Table 2:** Locus-wise and average gene diversity indices

Locus	Ht	Hs	G_ST_
D12S83	0.839997	0.805433	0.041147
D13S218	0.667397	0.614723	0.078924
D12S78	0.910758	0.871206	0.043428
D13S217	0.806758	0.754969	0.064194
D12S1659	0.566092	0.535312	0.054373
D13S285	0.844404	0.815226	0.034555
D13S170	0.866298	0.843637	0.026158
D12S1723	0.749630	0.721673	0.037294
D13S175	0.608139	0.547949	0.098974
D13S263	0.824989	0.781029	0.053286
D12S346	0.864446	0.828345	0.041761
D12S1617	0.844413	0.786153	0.068994
All loci	0.782777	0.742138	0.051916

**Figure 2:**
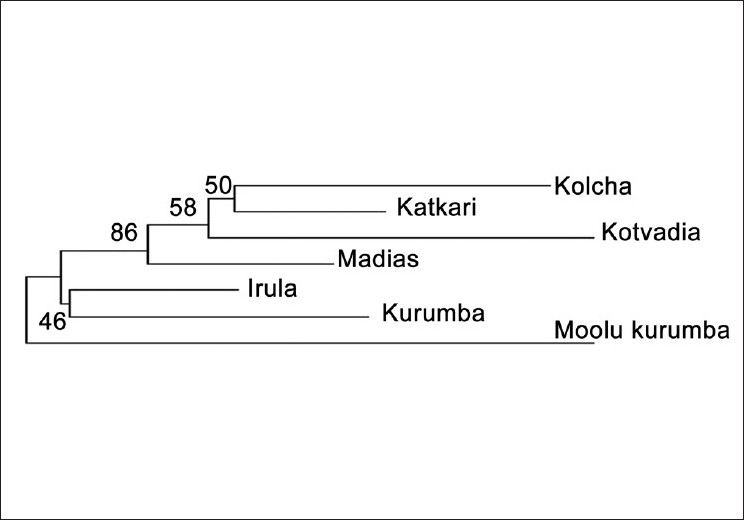
Neighbor-joining tree based on DA distances depicting genomic affinities among seven tribal population groups of India

**Figure 3:**
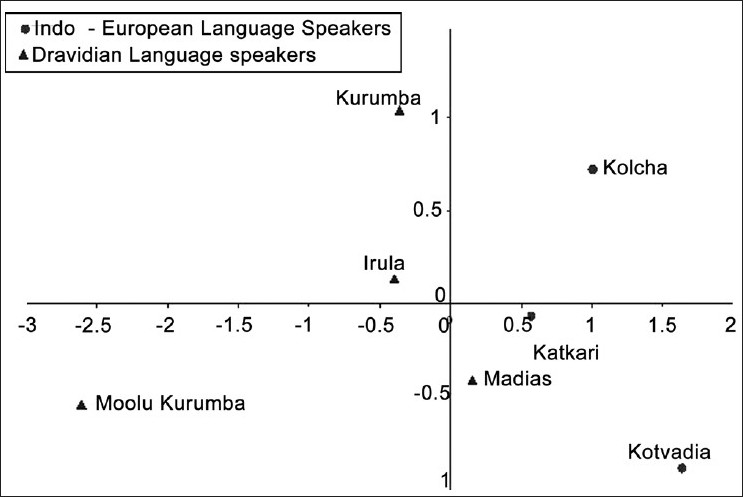
Bidimensional plot of tribal populations based on multidimensional scaling of the Nei's DA distance matrix

Pair-wise genetic distances between the study populations were computed from the allele frequencies of the 12 loci [Table 3] and an unrooted NJ tree was constructed from the distance matrix [Figure 2]. The study populations grouped themselves into two broad clusters in the tree, one formed by Kolchas, Katkaris and Kotvadias, the Indo-European-speaking tribal groups of Gujarat and Maharashtra along with Madias that are a Dravidian-speaking tribe but living in Maharashtra, and the other by Irulas and Kurumbas from Tamil Nadu, belonging to the Dravidian linguistic family. The Dravidian-speaking Moolu Kurumba tribe from the Nilgiris, Tamil Nadu in South India stand as an outlier distinctly separated from the rest with a relatively long branch. The NJ tree has low bootstrap values and so the relationships shown in the tree might not be highly reliable. A better way of visualization of interpopulation relationships can be achieved by a bivariate plot of populations based on multidimensional scaling of the Nei's DA distance matrix. The relative position of the populations in the multivariate space is depicted in Figure 3, in which the tribal groups of Maharashtra and Gujarat were placed in the right two quadrants whereas the tribes from Tamil Nadu were placed on the left quadrants, the outlier position of Moolu kurumba being unmistakably depicted.

**Table 3:** Matrix of genetic distances between the pairs of populations studied

	Kotvadia	Kolcha	Irula	Kurumba	Moolu kurumba	Katkari	Madias
Kotvadia	0.000						
Kolcha	0.158	0.000					
Irula	0.168	0.141	0.000				
Kurumba	0.180	0.152	0.098	0.000			
Moolu Kurumba	0.256	0.221	0.166	0.195	0.000		
Katkari	0.118	0.099	0.095	0.130	0.202	0.000	
Madias	0.122	0.132	0.092	0.137	0.178	0.094	0.000

AMOVA was performed, grouping the tribes according to the state to test whether geographically closer tribes are also genetically closer [Table 4]. Genetic differentiation between these tribal populations within the geographic groups is found to be significant and larger (F_ST_ value 0.03) when compared with that among the geographic groups, which is negligible and non-significant. Similar results were obtained when AMOVA was performed based on the linguistic groupings. Genetic differentiation within the Linguistic group was found to be significant compared with the non-significant value for genetic differentiation between the two linguistic groups.

**Table 4:** Analysis of molecular variance of the 12 STR loci

Source of variation	Population structure based on geographic groups				Population structure based on linguistic groups			
	d.f.	Sum of squares	Variance components	Percentage of variation	d.f.	Sum of squares	Variance components	Percentage of variation
Among groups	2	26.18	0.030	0.64	1	17.31	0.049	1.03
Among populations within groups	4	38.57	0.116	2.44	5	47.44	0.111	2.34
Within populations	313	1436.43	4.589	96.92	313	1436.43	4.589	96.63
Total	319	1501.18	4.735		319	1501.18	4.749	
Fixation indices	FST: 0.03079*				FST: 0.03373*			
	FSC: 0.02455*				FSC: 0.02368*			
	FCT: 0.00640				FCT: 0.01029			

* Significance at *P* ≤ 0.05

Mantel correlations between genetic distance and geographical or linguistic distances were not significant. The partial Mantel correlation was significant neither between genetic and geographical (*P* = 0.30) nor between genetic and linguistic distances (*P* = 0.58), respectively, controlling for linguistic and geographic distances.

## Discussion

The immense cultural, linguistic and ethnic diversity in the Indian population, which has crossed one billion, offers tremendous scope for genetic diversity studies in the country. Microsatellites are STRs composed of a core unit of one to five bases and are considered to be highly informative markers in the various fields of modern genetics. The dinucleotide microsatellite markers are known to be selectively neutral in nature. Therefore, observed variations in the allele frequencies could be due to random genetic drift or admixture. Because the populations under study have generally remained endogamous, similarities of allele frequencies among them are probably a reflection of their common ancestry.[8]

These populations show high levels of average heterozygosity (about 74%), suggesting high within-population diversity at these 12 loci. The average G_ST_ is observed to be 5.2%, suggesting a significant amount of inter-tribal differentiation. Further, this G_ST_ value is much higher than that observed for the traditional markers (1.5%) among the Indian populations. Based on STR loci, previous studies have reported a relatively high G_ST_ value among the Indian populations representing northern, eastern and northeast regions[17,18] as compared to the G_ST_ value based on STR, VNTR and other DNA marker loci among the subcastes of Golla from Southern Andhra Pradesh,[19] Bhargavas, Chaturvedis and Brahmins of North India[20,21] and also the tribal populations of Madhya Pradesh[8] and Orissa.[22] The average G_ST_ value calculated for three STR loci common to 23 Indian populations ranged from 3.2% among the Golla sub castes at the local level to 6.7% for all the 23 groups representing different regions of India.[23] Based on the 12 mirosatellite loci, G_ST_ value for the tribal populations of the present study is quite high as compared with the other continental populations (G_ST_ < 2%) such as Africans, Caucasians and Mongoloids.[24,25] This could be due to strict endogamy and small population sizes, which might have led to rapid genetic differentiation.

The genomic affinities among the groups studied [Figures 2 and 3] indicate that Katkaris, Kolchas and Kotvadias, the Indo-European-speaking tribes are closer to each other while the Madias (Dravidian-speaking tribe) join this cluster as an outer element. Although Madias and Katkaris are from the same state of Maharashtra, the two are from places that are geographically wide apart and also belong to different linguistic groups. On the other hand, Kolcha and Kotvadia from Gujarat are geographically close to Katkaris from Maharashtra. The genetic affinities between the tribes Kolcha, Kotvadia and Katkaris can thus be explained in terms of linguistic affiliation and geographic proximity. Irulas and Kurambas, both Dravidian-speaking tribes, are also genetically closer to each other. On the other hand, Moolu Kurumba, which is also a Dravidian-speaking tribe of Tamil Nadu, do not cluster with Irulas and Kurumbas. The samples for three Dravidian-speaking tribes Irula, Kurumba and Moolu Kurumba have been collected from the same place. Thus, for the Dravidian-speaking tribes, neither the linguistic affiliation nor the geographical proximity explain the population relationships. The position of Moolu Kurumba is hard to explain as, linguistically, Kurumba and Moolu Kurumba are closest to each other. The much longer branch length for Moolu Kurumba in the NJ tree probably indicates earlier separation and/or distinct origin as compared with the other Dravidian-speaking tribal populations. Although Madia also belongs to the Dravidian linguistic group, it is not close, linguistically, to either Irula or Kurumba and Moolu Kurumba. This might be the reason why we find that, genetically, Madia is not close to other Dravidian groups. Previous studies on the tribal groups like Katkaris and Irulas for other STR markers reported that these tribes are genetically distinct from other tribal groups.[26,27]

The analysis of molecular variance reveals that the extent of genetic differentiation among the states or among linguistic families is not significant although the genetic differentiation between populations within the states or within the linguistic family was significant, suggesting that the current geographic and/or linguistic boundaries are not significant determinants of genetic variation in these tribal populations. The political state boundaries in India may not be an actual indication of geographical and/or genetic barriers. MANTEL correlation and partial correlation analysis based on actual geographical distances also did not reveal any significant association between genetic and either linguistic or geographic distance.

The results based on just 12 loci might be sometimes misleading as some of the allele frequencies might have been highly affected by stochastic variation considering the limitation of small sample sizes. Nevertheless, these 12 STR loci exhibit a high discriminatory power. The PD is found to be in the range of 0.50-0.96. The high values of PD validate the utility of these markers in forensic identification as well. Further, earlier studies supported the utility of the STR markers in population discrimination both at the local and at the regional levels.[19,28] The large number of segregating alleles and the high value of heterozygosity further support the utility of these STR loci in the context of Indian populations for carrying out population genetic studies, linkage analysis and for forensic purposes.
